# Suppression of Virulent Porcine Epidemic Diarrhea Virus Proliferation by the PI3K/Akt/GSK-3α/β Pathway

**DOI:** 10.1371/journal.pone.0161508

**Published:** 2016-08-25

**Authors:** Ning Kong, Yongguang Wu, Qiong Meng, Zhongze Wang, Yewen Zuo, Xi Pan, Wu Tong, Hao Zheng, Guoxin Li, Shen Yang, Hai Yu, En-min Zhou, Tongling Shan, Guangzhi Tong

**Affiliations:** 1 Department of Swine Infectious Disease, Shanghai Veterinary Research Institute, Chinese Academy of Agricultural Sciences, Shanghai, China; 2 Jiangsu Co-innovation Center for Prevention and Control of Important Animal Infectious Diseases and Zoonoses, Yangzhou, 225009, China; 3 Department of Preventive Veterinary Medicine, College of Veterinary Medicine, Northwest A&F University, Yangling, Shaanxi, 712100, China; 4 Department of zoology, College of Life Science, Xinyang Normal University, Xinyang, Henan, 464000, China; Sun Yat-Sen University, CHINA

## Abstract

Porcine epidemic diarrhea virus (PEDV) has recently caused high mortality in suckling piglets with subsequent large economic losses to the swine industry. Many intracellular signaling pathways, including the phosphatidylinositol 3-kinase (PI3K)/Akt pathway, are activated by viral infection. The PI3K/Akt pathway is an important cellular pathway that has been shown to be required for virus replication. In the present study, we found that the PEDV JS-2013 strain activated Akt in Vero cells at early (5–15 min) and late stages (8–10 h) of infection. Inhibiting PI3K, an upstream activator of Akt, enhanced PEDV replication. Inhibiting GSK-3α/β, one of the downstream effectors of PI3K/Akt pathway and regulated by Akt during PEDV infected Vero cells, also enhanced PEDV replication. Collectively, our data suggest that PI3K/Akt/GSK-3α/β signaling pathway is activated by PEDV and functions in inhibiting PEDV replication.

## Introduction

Porcine epidemic diarrhea virus (PEDV) is an enveloped, single-stranded, positive-sense RNA virus belonging to the *Alphacoronavirus* genus in the coronavirus family. PEDV causes porcine epidemic diarrhea (PED), characterized by severe watery diarrhea, vomiting, dehydration, and significant mortality in suckling piglets [1–3]. The genome is approximately 28,000 bp and encodes at least seven open reading frames (ORFs), including the replicase polyproteins 1a and 1b, ORF3, spike (S), envelope (E), membrane (M), and nucleocapsid (N) proteins [4,5]. PED was first reported in Belgium at the United Kingdom in 1971 [6] and subsequently detected in many European and Asian countries [7–10]. Large outbreaks of PED caused by a PEDV variant strain in Chinese swine farms have caused large economic losses [11–13]. In 2013, a PEDV variant emerged and negatively impacted the United States swine industry [14–16].

The phosphatidylinositol-3-kinase (PI3K)/Akt signaling pathway regulates many cellular functions, including cell growth, cell proliferation, inflammation, and cell survival/apoptosis [17–19]. Activation of PI3K by cytokines, growth factors, or virus binding to cell surface receptors leads to the generation of phosphatidylinositol-3, 4, 5-triphosphate (PIP3). PIP3 recruits Akt to the plasma membrane and activates Akt by phosphorylation [20]. Activated Akt induces the phosphorylation of numerous signaling proteins, including FoxO1, p53, mTOR, Bad, and glycogen synthase kinase-3 (GSK-3) [21–25]. The PI3K/Akt pathway affects the replication of many viruses, including human immunodeficiency virus (HIV), influenza virus, reovirus, herpesvirus, severe acute respiratory syndrome coronavirus (SARS-CoV), and the porcine reproductive and respiratory syndrome virus (PRRSV) [23,24,26–30].

Glycogen synthase kinase-3 (GSK-3) is a serine/threonine protein kinase that is expressed ubiquitously in most mammalian cells. GSK-3αand GSK-3βare the two isoforms of the GSK-3 [31]. Unlike most proteinkinases, GSK-3 is actived in unstimulated resting cells and the activation of which is negatively regulated by phosphorylation [32]. The phosphorylation of GSK-3α(on Ser21) and GSK-3β(on Ser 9) is mediated by Akt [33], P70S6K [34], p90^RSK^ [35], PKC isoforms [36], and PKA [37]. GSK-3 plays an important role in regulating innate immune responses by regulating the activity of transcription factors such as NF-κB, NFAT and STATs [38–40]. In innate immune cells, GSK-3 augments the production of pro-inflammatory cytokines and suppresses anti-inflammatory cytokine production [38]. In the present study, we found that PEDV activated the PI3K/Akt pathway in Vero cells and that blocking PI3K activation promoted PEDV replication. Phosphorylation of GSK-3 a downstream target of PI3K/Akt, was increased by PEDV infection and blocking GSK-3 phosphorylation enhanced PEDV replication.

## Materials and Methods

### Viruses, cell cultures and reagents

The virulent PEDV strain JS-2013 used in this study was stored in Shanghai Veterinary Research Institute, Chinese Academy of Agricultural Sciences, which was isolated from the intestine of a suckling piglet with acute diarrhea in the region of Jiangsu province, China, in 2013. The virus stock was prepared in African green monkey kidney (Vero) cells, and stored at -80°C until use. Vero cells were maintained in Dulbecco’s modified Eagle’s medium (DMEM) (Thermo Fisher Scientific Inc, Waltham, USA) containing 10% fetal bovine serum (FBS) (Thermo Fisher Scientific Inc, Waltham, USA) at 37°C containing 5% CO_2_.

Antibodies specific for Akt, phospho-Akt (Ser473), phospho-GSK-3α/β(S21/S9), phospho-mTOR (S2448), phospho-Bad (S136) and mouse monoclonal β-actin were purchased from Cell Signaling Technology, Inc. (Beverly, MA, USA). Mouse anti-p53 antibody was kindly provided by Prof. Zhiyong Ma (Shanghai Veterinary Research Institute, Chinese Academy of Agricultural Sciences, Shanghai, China). Horseradish peroxidase (HRP)-conjugated anti-mouse IgG and anti-rabbit IgG were purchased from Proteintech Group, Inc. (Chicago, USA). The PI3K-specific inhibitor LY294002 was purchased from Cell Signaling Technology, Inc. (Beverly, MA, USA), and diluted to 10 mM in DMSO. The GSK-3α/βinhibitor CHIR-99021 was purchased from Selleck Chemicals (Houston, USA). The monoclonal antibody raised against the N protein of PEDV JS-2013 was prepared by our laboratory [41].

### Virus infection

Vero cells were grown to 90% confluence in six-well culture dishes and inoculated with PEDV JS-2013 at a multiplicity of infection (MOI) of 1 in the presence of 5 μg/ml trypsin (Thermo Fisher Scientific Inc, Waltham, USA). One hour post-infection (p.i.), Vero cells were washed three times with serum-free DMEM, and then cultured in fresh DMEM containing 5 μg/ml trypsin until the cells were harvest for analysis. Viral propagation was measured by using a monoclonal antibody raised against the N protein of PEDV JS-2013 in an indirect immunofluorescence assay (IFA).

In some experiments, Vero cells were pre-treated with DMSO or with 10 μM of the PI3K inhibitor LY294002 (dissolved in DMSO) one hour prior to PEDV infection. After virus adsorption at 37°C for 1 h, cells were cultured in fresh DMEM containing 5 μg/ml trypsin and LY294002. DMEM containing 5 μg/ml trypsin and DMSO (0.1%, v/v) was used for mock treatment. The cells were collected at 8 h post infection.

For experiments involving UV-inactivated viruses, PEDV viral stocks were dispersed in 100-mm tissue culture dishes and irradiated under a UV lamp (20 W) for 30 min. UV-irradiated viruses were tested for lack of infectivity by titration on Vero cells. UV-inactivated viruses at an MOI of 1 were then used in experiments designed to detect phosphorylated Akt within 1 h p.i. as indicated.

### Indirect immunofluorescence assay

At the indicated hours postinfection, vero cells were fixed with cold 4% paraformaldehyde for 15 min at room temperature, washed with phosphate-buffered saline (PBS, pH 7.4), and then permeated with 0.5% Triton X-100 for 5 min at room temperature. After three washes with PBS, the cells were blocked with 5% bovine serum albumin for 1 h and then incubated with mouse monoclonal antibody against PEDV N protein (1:2000) for 1 h at 37°C. The cells were then treated with donkey anti-mouse Alexa Fluor 488 (Thermo Fisher Scientific Inc, Waltham, USA) for 1 h at 37°C and then treated with 4’, 6-diamidino-2-phenylindole (DAPI) for 5 min at room temperature. Following washing, fluorescence images were captured by using a Nikon inverted fluorescence microscope.

### Western blotting

After virus infection, cells were washed twice with cold PBS and lysed on ice in RIPA lysis buffer (25 mM Tris-HCl, pH 7.6, 150 mM sodium chloride, 1% NP-40, 1% sodium deoxycholate, 0.1% SDS) according to the manufacturer’s instructions. After separation using sodium dodecyl sulfate polyacrylamide gel electrophoresis (SDS-PAGE), proteins were transferred to nitrocellulose membranes (Millipore, Billerica, MA, USA). The membranes were blocked for one hour at room temperature (RT) using non-fat dry milk solution (5% non-fat dry milk in Tris-buffered saline containing 0.2% Tween-20). Blots were incubated 1 h at RT with primary antibodies. After 3 washes with TBS containing 0.1% Tween-20 (TBS-T buffer), the membranes were incubated 1 h at RT with secondary antibody (conjugated horseradish peroxidase) and detected using enhanced chemiluminescence (ECL) (Thermo Fisher Scientific Inc, Waltham, USA). β-actin protein abundance was used for data normalization.

### Virus titration

The virus titer was determined by the 50% tissue culture infectious dose (TCID_50_). Vero cells monolayers grown in 96 well plates and inoculated with 100 μl of 10-fold diluted cell culture supernatants. After 48 h of incubation, the CPE was observed by Nikon inverted microscope. The TCID_50_ was calculated using the method of Reed and Munch.

### RNA extraction and real-time PCR

Total RNA was extracted using the QIAamp^®^ Viral RNA Mini Kit (QIAGEN, Hilden, Germany) according to the manufacturer’s protocol. RNA was reverse transcribed to cDNA using PrimeScript™ 1st Strand cDNA Synthesis Kit (TAKARA BIO INC., Dalian, Japan). PCR products were purified and cloned into the pcDNA™3.1(+) plasmids and used to establish a standard curve. Absolute quantification Real-time PCR analysis of the PEDV N protein gene was performed using the primers (forward: 5’-GAGGGTGTTTTCTGGGTTG-3’; Reverse: 5’-CGTGAAGTAGGAGGTGTGTTAG-3’) and SYBR Green PCR Master Mix (TAKARA BIO INC., Dalian, Japan) in an ABI StepOne plus Real-Time PCR System (Applied Biosystems, Inc., USA). β-actin (forward: 5’- TCCCTGGAGAAGAGCTACGA-3’. Reverse: 5’-AGCACTGTGTTGGCGTACAG-3’) was used for normalization in gene expression analysis.

### Statistical analysis

Statistical analysis was performed using Prism 5.0 software (GraphPad). Significance was determined by using two-tailed independent student t-tests. Statistical significance: *p < 0.05, **p < 0.001.

## Results

### PEDV infection activates the PI3K/Akt pathway

**Fig 1:**
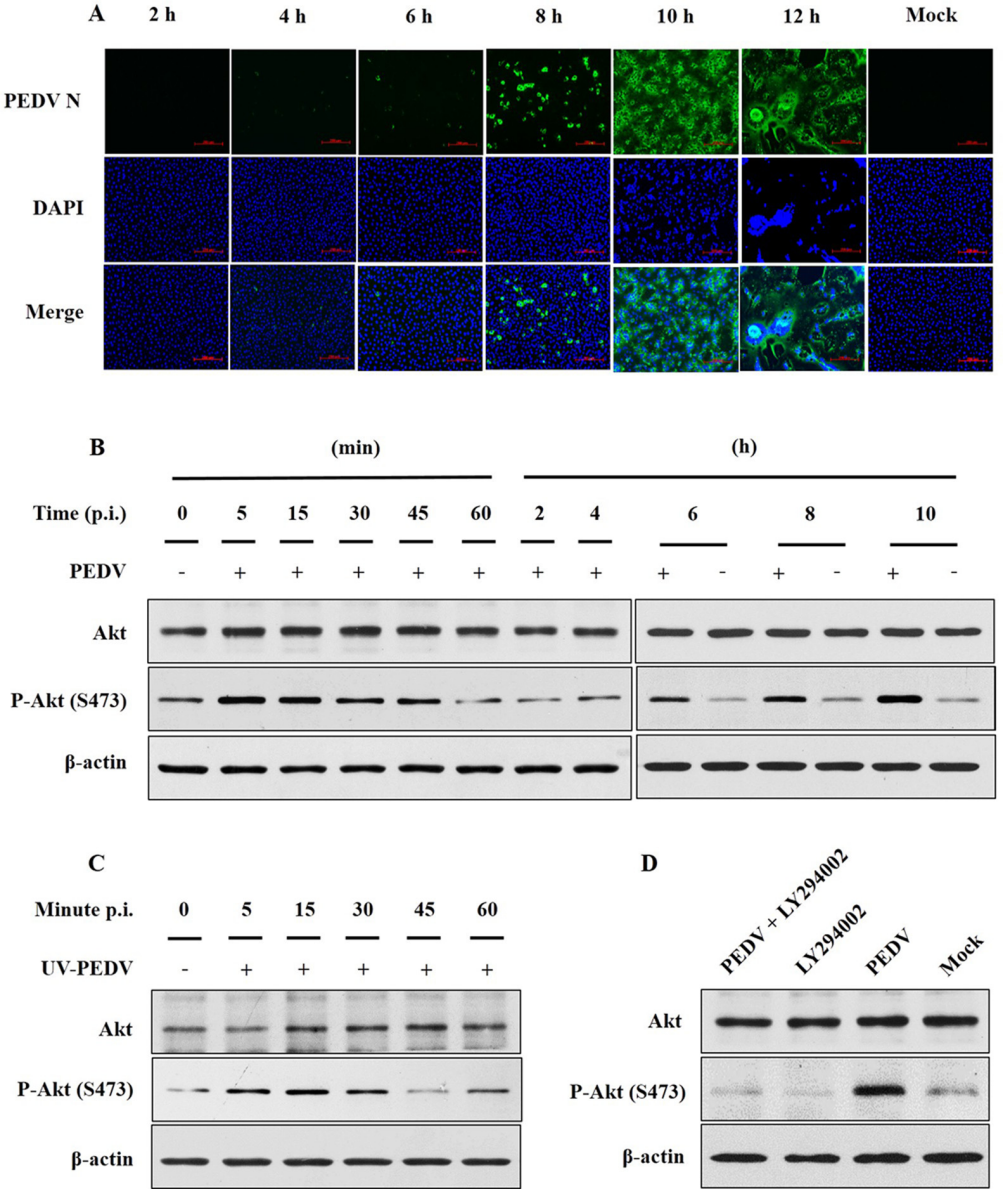
PEDV JS-2013 activates the PI3K/Akt pathway. **A.** Vero cells were infected with PEDV JS-2013 at an MOI of 1 and processed for immunofluorescence microscopy at the indicated time points, using a monoclonal N protein antibody and DAPI. **B.** Cells were harvested at the indicated time points and analyzed using Western blotting. **C.** Infection with UV-inactivated PEDV. **D.** Vero cells were pretreated with LY294002 (10 μM) 1 h prior to PEDV infection. At 8 h p.i., cells were harvested and analyzed using Western blotting. All experiments were repeated three times and representative results are shown.

Vero cells were infected with the PEDV JS-2013 strain at an MOI of 1. Viral propagation was measured by using an indirect immunofluorescence assay (IFA) at 2–12 h after PEDV infection. PEDV N protein expression was detected initially at 6 h p.i. and increased until 12 h p.i. (**Fig 1A**).

We then analyzed Akt activation as a function of PEDV infection by monitoring Akt phosphorylation using Western blotting. We observed increased Akt phosphorylation at early infection times (5–15 min p.i.; **Fig 1B**). Phosphorylated Akt levels returned to baseline at 1 h p.i., but then increased from 6–10 h p.i. (**Fig 1B**). UV-irradiated PEDV also increased Akt phosphorylation at early infection times (**Fig 1C**). Treating cells with the PI3K inhibitor LY294002 during PEDV infection abolished the increase in Akt phosphorylation, indicating that Akt phosphorylation was PI3K-dependent (**Fig 1D**).

### Inhibiting the PI3K/Akt pathway promotes PEDV replication

**Fig 2:**
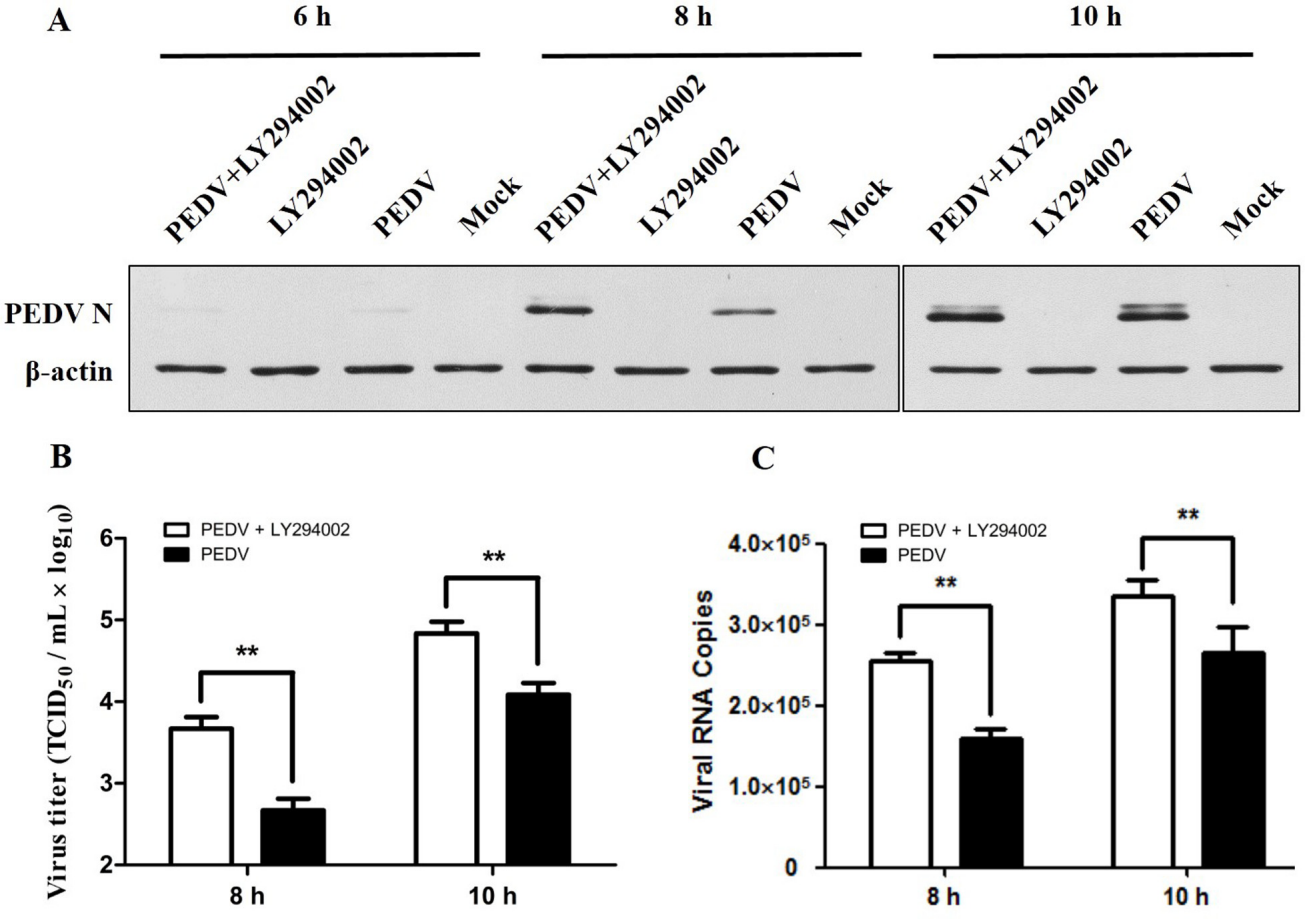
Inhibition of the PI3K/Akt pathway promotes PEDV replication. **A.** Vero cells were pretreated with LY294002 (10 μM) 1 h prior to PEDV infection. Cell lysates were prepared after 6–10 h of infection and then analyzed using Western blotting. **B.** Quantification of viral titers using TCID_50_ assays. **C.** Quantification of viral RNA copies using real-time PCR. Quantitative data are shown as mean ± SD (**p < 0.001) of three independent experiments.

We treated Vero cells with LY294002 and then infected them with PEDV (MOI = 1). As measured based on Western blotting (**Fig 2A**), quantification of the TCID_50_ (**Fig 2B**), and real-time PCR (**Fig 2C**), PEDV titers were significantly increased in Vero cells treated with LY294002 at 8–10 h p.i., indicating that blocking the PI3K/Akt signaling pathway promotes virus replication.

### PEDV activates GSK-3α/β

**Fig 3:**
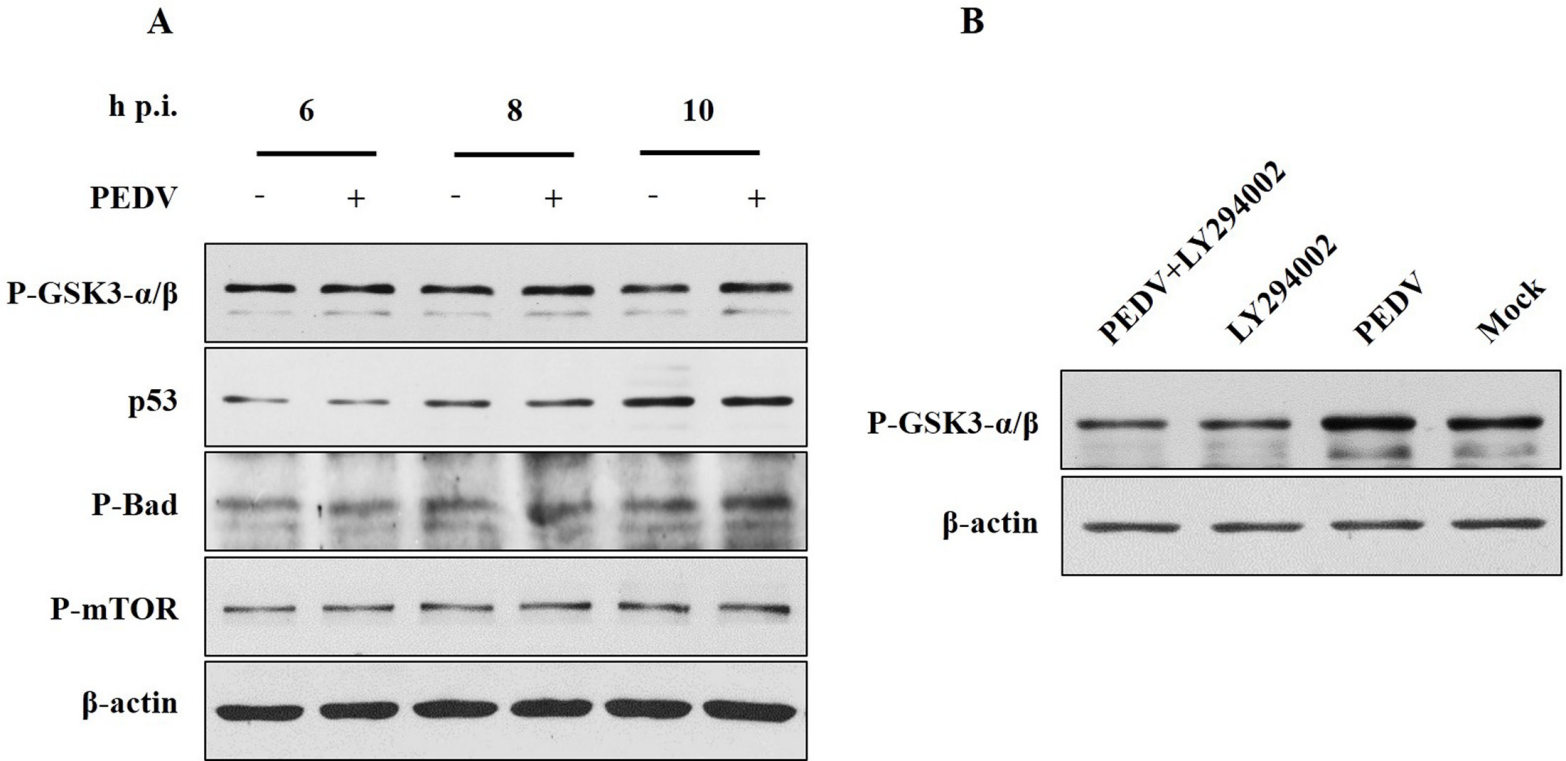
PEDV affects GSK-3α/β phosphorylation. **A.** Vero cells were infected with PEDV at an MOI of 1 and harvested for Western blotting at the indicated times after infection. **B.** Vero cells were pretreated with LY294002 (10 μM) 1 h prior to PEDV infection. Cell lysates were prepared after 8 h of infection and then analyzed using Western blotting. All the experiments were repeated three times and representative results are shown.

To analyze further the mechanism of PI3K/Akt pathway in regulating PEDV replication, several downstream targets of Akt in PEDV-infected cells were examined by using Western blotting. Compared to the mock-infected control, PEDV infection caused a significant increase in GSK-3α/βphosphorylation, but did not affect Bad or mTOR phosphorylation, nor did it affect p53 abundance (**Fig 3A**). To determine whether GSK-3α/βphosphorylation was induced by PEDV through the PI3K/Akt pathway, Vero cells pre-treated with LY294002 were infected with PEDV, and then analyzed. LY294002 treatment decreased GSK-3α/βphosphorylation, as compared with the mock treatment (**Fig 3B**).

### GSK-3α/β inhibits PEDV replication

**Fig 4:**
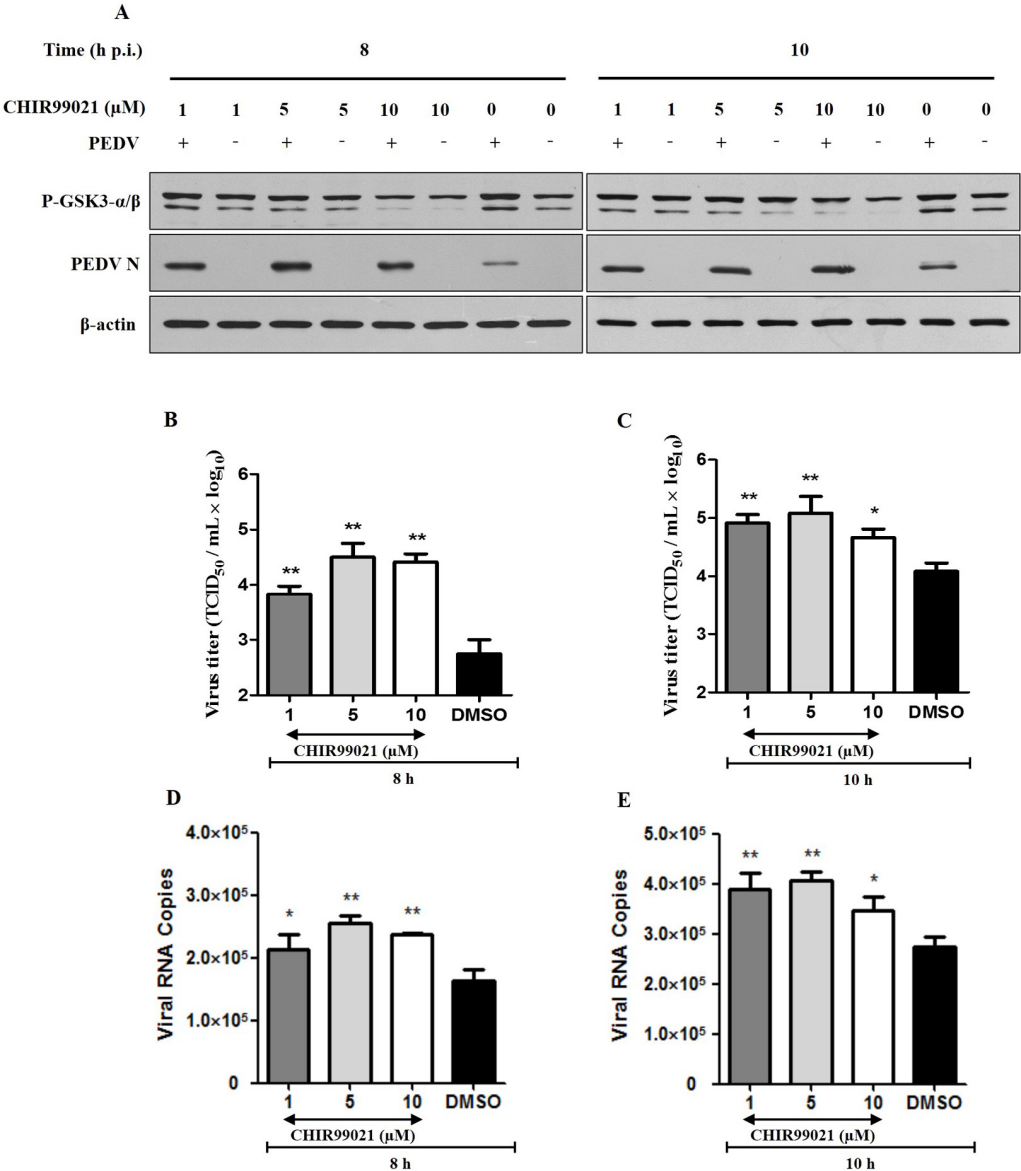
Inhibition of GSK-3α/βphosphorylation promotes PEDV replication. **A.** Vero cells were pretreated with the indicated concentrations of CHIR-99021 for 1 h and then infected with PEDV (MOI = 1) for 8 or 10 h. Cell lysates were prepared and analyzed using Western blotting. **B.** Quantification of viral titers using TCID_50_ assays after 8 h PEDV infection. **C.** Quantification of viral titers using TCID_50_ assays after 10 h PEDV infection. **D.** Quantification of viral RNA copies using real-time PCR after 8 h PEDV infection. **E.** Quantification of viral RNA copies using real-time PCR after 10 h PEDV infection. Quantitative data are shown as mean ± SD (*p < 0.05, **p < 0.001) of three independent experiments.

We pre-treated Vero cells with the GSK-3α/βinhibitor CHIR-99021, infected the cells with PEDV, and analyzed PEDV replication from 8–10 h p.i. CHIR-99021 pre-treatment (1–10 μM) decreased GSK-3α/βphosphorylation, with a concomitant increase in PEDV N protein abundance (**Fig 4A**). Experiments in which PEDV titers and viral RNA copies were quantified corroborated the Western blotting data (**Fig 4B–4E**).

## Discussion

In this study, we characterized the role of the PI3K/Akt/GSK-3 signaling pathway in controlling PEDV JS-2013 infection in Vero cells. We observed PI3K-dependent Akt phosphorylation at both the early and late stages of PEDV infection. Inhibiting Akt phosphorylation by the PI3K inhibitor LY294002 significantly increased viral replication in Vero cells. Furthermore, the PI3K/Akt downstream effector GSK-3α/βwas activated during PEDV infection and inhibiting the activation of GSK-3α/β by CHIR-99021 increased viral replication. These findings demonstrate that the activation of the PI3K/Akt/GSK-3 signaling pathway is important for inhibiting PEDV replication.

Viral infection can impact the activity of many intracellular signaling pathways, including the PI3K/Akt pathway [42,43]. The PI3K/Akt signaling pathway plays an important role in cell survival and virus replication. A previous report showed that PRRSV and UV-PRRSV induce early transient Akt activation in porcine alveolar macrophages and Marc-145 cells to facilitate virus entry [28]. The vaccinia mature virus infected Hela cells triggers the activation of PI3K/Akt pathway, and inhibited the PI3K activation reducing virus entry in an integrin β1-dependent manner [44]. Another report showed that the influenza A virus NS1 protein activates the PI3K/Akt pathway by direct interaction [45]. Our study observed that PEDV stimulated the activation of PI3K/Akt pathway in Vero cells at both the early (5–15 min p.i.) and late (6–10 h p.i.) stage (**Fig 1B**). We also found that akt phosphorylation was also enhanced by UV-inactivated PEDV (**Fig 1C**). Taking these findings together, we conclude that PI3K/Akt pathway could be activated in virus replication and virus invasion. It could be that PI3K/Akt activation not only triggered dependent upon virus replication-competent particles but also triggered by PEDV-host receptor interactions.

Previous reports indicated that activation of the PI3K/Akt pathway can increase influenza virus and human papillomavirus replication [26,46]. Another report indicated that activation of the PI3K/Akt pathway suppress reovirus virus replication [24]. Here we showed that PEDV activates the PI3K/Akt pathway and inhibiting PI3K activation promoted PEDV replication in Vero cells (**Fig 2**).

Several downstream targets of Akt that play important roles in viral infections have been identified and include mTOR, p53, Bad, and GSK-3 [24,43,47,48]. Some studies have reported that the phosphorylation of mTOR and Bad regulate cell survival and effect virus infection. The expression of p53 was induced by PI3K/Akt pathway after reovirus infection and inhibited virus replication [24]. GSK-3 regulates innate immune responses, transcription factors, and cytokine expression [47,49–51]. We found both that PEDV affected GSK-3α/βphosphorylation in late stages of infection (**Fig 3)** and the phosphorylation of GSK-3α/β was regulated by Akt. Inhibited GSK-3α/β phosphorylation increased PEDV replication in Vero cells (**Fig 4**). The precise molecular mechanism by which GSK-3 regulates PEDV replication requires further investigation.
